# Migraine, dyspepsia, and Helicobacter pylori: Zeroing in on the culprit

**Published:** 2019-01-05

**Authors:** Nayereh Akbari, Ahmad Hormati, Ehsan Sharifipour, Seyed Amir Hejazi, Fatemeh Jafari, Seyed Ali Mousavi-Aghdas, Samad EJ Golzari

**Affiliations:** 1 Neuroscience Research Center, Qom University of Medical Sciences, Qom, Iran; 2 Gastroenterology and Hepatology Research Center, Qom University of Medical Sciences, Qom, Iran; 3 Student Research Committee, Tabriz University of Medical Sciences, Tabriz, Iran; 4 Research Center for Evidence Based Medicine, Tabriz University of Medical Sciences, Tabriz, Iran; 5 Road Traffic Injury Research Center, Health Management and Safety Promotion Research Institute, Tabriz University of Medical Sciences, Tabriz, Iran

**Keywords:** Migraine, Dyspepsia, Helicobacter Pylori, Peptic Ulcer

## Abstract

**Background:** Numerous studies have evaluated the impact of Helicobacter pylori (H. pylori) eradication on the number, severity, and recurrence of migraine attacks. But the association of migraine, H. pylori, and gastrointestinal (GI) presentation is challenging. The aim of the current study was to investigate the correlation between migraine, H. pylori, and peptic ulcers among patients with dyspepsia undergoing upper GI endoscopy.

**Methods:** 305 patients with dyspepsia referring to our endoscopy ward, Shahid Beheshti Hospital affiliated to Qom University of Medical Sciences, Qom, Iran, for upper GI endoscopy filled out the study questionnaire. If a patient was experiencing headaches and the migraine was confirmed by neurologists, he/she was asked to answer the questions related to migraine, which were prepared exactly from Migraine Disability Assessment (MIDAS) questionnaire. The relation between migraine and confirmed H. pylori contamination was investigated using statistical models.

**Results:** Of all the 305 patients, 133 (43.6%) had confirmed episodic migraine headaches (MHs) and 177 patients (58.04%) had positive RUT for confirming H. pylori contamination, of which 123 (69.5%) had confirmed migraine. 52 (17.0%) had duodenal peptic ulcer(s), of which, 49 (94.2%) had a positive rapid urease test (RUT) (P < 0.001). 20 (6.5%) of all patients had the gastric peptic ulcer(s) which did not have a significant relation with H. pylori contamination. There was a significant relationship between the peptic ulcer site and migraine. In total, 177 patients (58.0%) had a positive RUT. History of migraine was significantly positive in those with positive H. Pylori contamination. Notably, multivariable analysis demonstrated a significant relation of H. pylori and migraine at younger ages.

**Conclusion:** The prevalence of H. pylori and migraine in patients with dyspepsia seems to be high. Moreover, there is a meaningful association between migraine, duodenal peptic ulcers, and H. pylori infection, too.

## Introduction

Migraine is a common episodic syndrome of unilateral throbbing headache accompanied by nausea, vomiting, and photophobia.^1^^,^^2^ Since migraine attacks are debilitating and prevalent, they have a huge economic burden on the society.^3^ The exact pathophysiology of migraine is unclear but inherited nature and also repeated triggers by exogenous and endogenous stimulants are of the important aspects of this disorder.^4^ Most frequent triggering factors are limited to stress, fatigue, fasting, alcohol, sleep deprivation, climatic conditions, and hormonal, visual, olfactory, and auditory stimuli.^5^ Also several mechanisms such as serotonergic pathways and pain mediators like calcitonin-gene-related peptide (CGRP) alterations and inflammation are believed to be involved in the process of triggering migraine onset.^6^^-^^9^

Helicobacter pylori (H. pylori) is a helical gram-negative bacterium involved in peptic ulcer disease, gastric adenocarcinoma, and lymphoma.^10^ The association between H. pylori and numerous extragastric diseases such as coronary artery diseases (CADs), insulin resistance, urticaria, rosacea, Parkinson’s disease (PD), idiopathic thrombocytopenic purpura (ITP), and iron deficiency anemia has been previously shown.^11^^,^^12^ Also some studies reported a significant correlation between H. pylori contamination and migraine, and demonstrated that after H. pylori eradication, migraine symptoms reduced in the majority of the studied patients.^13^^-^^16^ Furthermore, numerous studies have shown the efficacy of H. pylori eradication on the number, severity, and recurrence of migraine attacks.^15^^,^^17^ On the other hand, some studies have demonstrated the protective role of H. pylori [especially, cytotoxin-associated gene A (cagA) positive strain] against reflux-induced esophagitis, Barrett’s esophagus, and possibly esophagogastric junction adenocarcinoma.^18^^-^^20^ Thus, careless eradication of H. pylori may be unbeneficial in asymptomatic patients.^21^ Altogether, the current data are limited about the exact correlation, especially regarding the relationship between the migraine and the peptic ulcers. In this study, we investigated the correlation between migraine and H. pylori contamination among patients with dyspepsia undergoing upper gastrointestinal (GI) endoscopy in a referral hospital in Iran.

## Materials and Methods

In this cross-sectional study, we assessed the correlation between H. pylori contamination and migraine among 305 patients undergoing upper endoscopy in a tertiary specialized center, Shahid Beheshti hospital, affiliated to Qom University of Medical Sciences, Qom, Iran. Patients were recruited from May 2016 to May 2017.

305 patients with refractory dyspepsia referring to our endoscopy ward for upper GI endoscopy filled out the study questionnaire. Patients having used antibiotics or proton-pump inhibitors (PPIs) in the last 2 weeks were excluded. The questionnaire consisted of sex, age, height, weight, occupation, education, family history of headaches, previous medical history (headaches, diabetes, ischemic heart diseases, respiratory diseases, chronic kidney diseases (CKDs), rheumatologic diseases, etc.). If a patient was experiencing headaches, he/she was referred to an experienced neurologist. If the diagnosis of MH (with or without aura) was confirmed by the expert neurologists using the International Classification of Headache Disorders-3^rd^ Edition (ICHD-3) (beta version).^1^

Patients with confirmed migraine were asked to answer the study questionnaire which were prepared exactly from the Migraine Disability Assessment (MIDAS) questionnaire with eleven-point pain scale.^22^^-^^23^ Questions related to findings from endoscopy (iron deficiency anemia, vomiting, epigastric pain, melena, hematemesis, rectorrhagia, dyspepsia, etc.), findings in esophagus (reflux, erosive esophagitis, stricture, varices, neoplasia, etc.), stomach, and duodenum (peptic ulcer, erosive gastritis, hernia, neoplasia, etc.), H. pylori contamination using rapid urease test (RUT) results, and laboratory results from liver function test [alanine aminotransferase (ALT), aspartate transferase (AST), alkaline phosphatase (ALP), and bilirubin)]. Two researchers assisted patients while answering the questionnaire. Primary outcome of the present study was to investigate the correlation between migraine with H. pylori contamination and peptic ulcers among patients with dyspepsia undergoing upper gastrointestinal endoscopy.

The current study was approved by the Ethical Committee of Qom University of Medical Sciences, in accordance with the Declaration of Helsinki and its later amendments. Written informed consent was obtained from all patients. Patients unwilling to participate in the study were excluded.

**Table 1:** Demographic characteristics of the study patients

**Variables**	**Minimum-Maximum**	**Mean **±** SD**
Age (year)	12-88	43.94 ± 15.13
Weight (kg)	37-155	66.91 ± 13.84
Height (cm)	100-192	164.03 ± 10.00
BMI (kg/m^2^)	13.10-47.00	26.04 ± 4.79
MH attack duration (hour)	0.5-72.0	15.49 ± 8.30
MH history (year)	0.5-30.0	4.88 ± 3.20
MH severity*	1-10	4.21 ± 4.04
MIDAS score	4-40	12.64 ± 8.12
MH Disability (days in one month)	0-7	0.94 ± 0.40

BMI: Body mass index; MH: Migraine headache; MIDAS: Migraine disability assessment; SD: Standard deviation

* Based on eleven-point pain scale

To present the data, we used descriptive statistical methods such as mean, standard deviation, and frequency. More precisely, independent Student’s t (for quantitative variables), Fisher’s exact (for categorical variables), and chi-square tests were used for data analysis. A P-value less than 0.05 was considered statistically significant. All of the statistical analyses were performed on IBM SPSS software (version 22, IBM Corp., Armonk, NY, USA).

## Results

A total number of 305 patients were included in our study. Demographic characteristics of the patients are presented in table 1. The female sex formed 60.3% of the entered population. 68.9% of the patients had education level of lower than diploma. Prevalence of migraine in patients with dyspepsia was 43.6% (133 patients, all suffering from episodic migraine without any chronic migraine) and this was significantly higher in female than male patients (48.9% vs. 35.5%, respectively) (P < 0.010). Of all the patients, 129 (42.3%) had a positive family history of headaches in their 1^st^ or 2^nd^ degree relatives. Detailed data related to MH of patients is demonstrated in table 1. 

In total, 177 patients (58.0%) had a positive RUT for confirming present H. pylori contamination. Of all patients with confirmed H. pylori, 123 patients (69.5%) had confirmed migraine, but of 128 non H. pylori contaminated patients, only 10 patients (3.27%) were suffered from migraine. The relation between H. pylori contamination and presence of migraine in patients with dyspepsia was significant (P < 0.001) (Table 2).

Furthermore, of all patients undergoing upper GI endoscopy, 52 (17.0%) patients had duodenal peptic ulcer(s), of which, 49 (94.2%) had a positive RUT (P < 0.001). 20 (6.5%) of all patients had gastric peptic ulcer(s) which did not have a significant relation with H. pylori contamination. Although the relation between H. pylori contamination with age and education was not significant, there was significant association between H. pylori contamination and positive family history of headaches (P < 0.001) (Table 3). 

Interestingly, the association between migraine and duodenal peptic ulcer was significant (P = 0.002); nevertheless, the association between migraine and gastric peptic ulcer was not significant (Table 4). Also, there was a significant association between migraine and positive family history of headaches, and migraine was significantly more prevalent in women (P < 0.001 and P < 0.050, respectively).

The relations of MH with age and education were not significant (Table 4). Notably, multivariable analysis demonstrated the significant relation of H. pylori and migraine at younger ages.

**Table 2:** The relation between Helicobacter pylori (H. pylori) contamination and migraine

**H. pylori contamination**	**Migraine**		**Total [n (%)]**
	**Positive [n (%)]**	**Negative [n (%)]**	
Positive	123 (40.32)	54 (17.70)	177 (58.04)
Negative	10 (3.27)	118 (38.68)	128 (41.96)
Total	133 (43.60)	172 (56.40)	-
P	< 0.001	-	-

H. pylori: Helicobacter pylori

**Table 3:** The relation between Helicobacter pylori (H. pylori) contamination and other variables

**Variables**		**H. pylori contamination**		**P**
		**Positive**	**Negative**	
Sex [n (%)]	Male	72 (59.5)	49 (40.5)	0.673
	Female	105 (57.1)	79 (42.9)	
Education [n (%)]	Below diploma	123 (58.6)	87 (41.4)	0.937
	Diploma	33 (58.9)	23 (41.1)	
	Associate	4 (66.7)	2 (33.3)	
	Bachelor	14 (51.9)	13 (48.1)	
	Master or above	3 (50.0)	3 (50.0)	
Family history of MHs [n (%)]	Yes	94 (72.9)	35 (27.1)	< 0.001
	No	83 (47.2)	93 (52.8)	
Duodenal ulcer [n (%)]	Yes	49 (94.2)	3 (5.8)	< 0.001
	No	128 (50.6)	125 (49.4)	
Gastric	Yes	13 (65.0)	7 (35.0)	0.514
Ulcer [n (%)]	No	164 (57.5)	121 (42.5)	
Age (year) (mean ± SD)		44.36 ± 13.18	43.36 ± 17.51	0.306

H. pylori: Helicobacter pylori; MH: Migraine headache; SD: Standard deviation

## Discussion

The current cross-sectional study demonstrated that migraine is a common complain among dyspeptic patients. Also, there is a meaningful correlation between migraine, duodenal peptic ulcers, and H. pylori.

It seems that there is an association between migraine and GI presentations (clinically or sub-clinically). In addition, recent studies addressed that H. Pylori infection had the association with some extra GI disorders such as migraine.^24^^-^^26^ Although some studies insist on the possible role of H. pylori infection in migraine precipitation, the others (spatially epidemiological sources) only emphasize the idea of a simple co-occurrence of these two.^16^^,^^21^^,^^27^

Results from a meta-analysis are also in consistence with our findings. In the mentioned study, a total number of 903 patients were included and it was shown that patients with migraine were more commonly contaminated with H. pylori.

At least one positive test for H. pylori infection including serology, RUT, mucosal biopsy, polymerase chain reaction (PCR), or urea breath test (UBT) was needed in this study.^28^ In our study, we used RUT because it is cost-effective and a positive RUT indicates present contamination.

**Table 4:** The relation between migraine headache (MH) and other variables

**Variables**		**Migraine**		**P**
		**Positive**	**Negative**	
Sex [n (%)]	Male	43 (35.5)	78 (64.5)	0.021
	Female	90 (48.9)	94 (51.1)	
Education [n (%)]	Below diploma	92 (43.3)	119 (56.7)	0.549
	Diploma	28 (50.0)	28 (50.0)	
	Associate	2 (33.3)	4 (66.7)	
	Bachelor	11 (40.7)	16 (59.3)	
	Master or above	1 (16.7)	5 (83.3)	
Family history of headaches [n (%)]	Yes	86 (66.7)	43 (33.3)	< 0.001
	No	47 (26.7)	129 (73.3)	
Duodenal ulcer [n (%)]	Yes	33 (63.5)	19 (36.5)	0.002
	No	100 (39.5)	153 (60.5)	
Gastric ulcer [n (%)]	Yes	9 (45.0)	11 (55.0)	0.897
	No	124 (43.5)	161 (56.5)	
H. pylori contamination [n (%)]	Yes	123 (69.5)	54 (30.5)	
	No	10 (7.8)	118 (92.2)	
Age (year) (mean ± SD)		42.70 ± 12.61	44.36 ± 16.79	0.398

H. pylori: Helicobacter pylori; SD: Standard deviation

Hosseinzadeh, et al. in a case-control study have also shown a significant relation between anti H. pylori immunoglobulin G (IgG) and immunoglobulin M (IgM) titers and severity of migraine.^29^ A study on childhood migraine has also suggested that H. pylori contamination is more common in this population. However, the study could not strongly recommend UBT for these patients.^30^ A study on 60 patients with migraine showed that those infected with H. pylori experienced less frequent and less severe migraine attacks after H. pylori eradication.^31^ Lee, et al. in a study used the clinical data warehouse method to analyze data regarding patients with different types of headaches and showed non-significant findings in keeping with higher prevalence of H. pylori contamination among migraine patients.^32^ Faraji, et al. in a double-blind clinical trial on 64 patients with migraine showed that H. pylori eradication caused a significantly larger decrease in the MIDAS score in contrast to the control group receiving placebo.^15^ Doulberis, et al. in a recent literature review demonstrated a higher prevalence of GI presentations among patients with migraine, although a clear pathophysiology remained unclear.^9^ On the other hand, Pinessi, et al. reported that the infection of H. pylori was not more common among patients with migraine compared to control patients.^33^

In our study, the relation between H. pylori contamination and presence of migraine in patients with dyspepsia was significant. Interestingly, migraine and duodenal peptic ulcers were significantly correlated, while there was no significant relation between gastric ulcers and migraine. This may be due to significant correlation between ulcer at the duodenum and H. pylori contamination in contrast to gastric ulcer which was not significantly correlated with H. pylori infection. This finding emphasizes the migraine association of H. pylori contamination with the difference in the site of peptic ulcers (stomach or duodenum). At this study, female sex was a risk factor for migraine, but age and education level were not correlated with migraine.

## Conclusion

The present study on 305 patients showed a significant correlation between dyspepsia, migraine, and H. pylori infection. The prevalence of H. pylori and migraine in patients with dyspepsia seems to be high, and a large percentage of migraine cases in these individuals are associated with H. pylori. Moreover, there was a significant relation between the peptic ulcer site and migraine, and duodenal ulcers were significantly related to migraine. Thus, migraine could be a disease beyond just brain involvement and H. pylori seems to be one of the factors having a meaningful association on this relation. Paying more attention to digestive problems in migraine and headache in patients with dyspepsia is beneficial as well as H. pylori eradication in these patients.

## Notes:

**How to cite this article:** Akbari N, Hormati A, Sharifipour E, Hejazi SA, Jafari F, Mousavi-Aghdas SA, et al. Migraine, dyspepsia, and Helicobacter pylori: Zeroing in on the culprit. Iran J Neurol 2019; 18(1): 19-24.
